# Verteporfin inhibits cell proliferation and induces apoptosis in different subtypes of breast cancer cell lines without light activation

**DOI:** 10.1186/s12885-020-07555-0

**Published:** 2020-10-29

**Authors:** Changran Wei, Xiangqi Li

**Affiliations:** 1 Shandong University of Traditional Chinese Medicine, Jinan, Shandong Province China; 2 Department of Breast Surgery, The Second Affiliated Hospital of Shandong First Medical University, No.706 TaiShan Road, TaiShan District, Tai’an, 271000 Shandong Province China

**Keywords:** Breast cancer, YAP, Verteporfin, Cell proliferation, Apoptosis

## Abstract

**Background:**

Breast cancer (BC) can be divided into five subtypes: Lumina1A, Lumina1B, HER-2 overexpression, Basal-like and Normal breast-like subtype, based on the differently expressed genes in breast cancer tissue. The Hippo signaling pathway plays an indispensable role in BC. The YAP gene is a terminal effector of Hippo pathway, and hyperactivation of YAP mediates tumorigenesis. As an inhibitor of YAP, non-photoactivated verteporfin (VP) can inhibit YAP-mediated tumor proliferation and angiogenesis by eliminating its interaction with TEAD. This study aimed to determine the effect and molecular mechanisms of VP-mediated inhibition of YAP in different subtypes of BC.

**Methods:**

Luminal A, Luminal B and Basal-like BC cells were cultivated in vitro to study effects of VP on proliferation and apoptosis of these three molecular BC subtypes.

**Results:**

Our experimental results showed that VP inhibited cell proliferation, YAP-TEAD interaction and expression of its downstream targets. VP also induced tumor cell apoptosis, and promoted the cleavage of Caspase-9 and PARP in the cells of various molecular subtypes of BC.

**Conclusion:**

These findings provide a basis for the use of VP as a potential anti-tumor therapeutic for BC by targeting the Hippo pathway effector YAP.

## Background

Breast cancer (BC) is a common malignant tumor with over 250,000 newly diagnosed cases reported every year [1]. The treatment regimen for BC depends primarily on the level of the estrogen receptor (ER), progesterone receptor (PR), human epidermal growth factor receptor-2 (HER-2) and Ki-67 in tumor patients. In addition to traditional surgery, the different subtypes of BC may require endocrine therapy, chemotherapy, radiotherapy, and biologically-targeted treatment [2, 3]. However, early metastasis and drug resistance have been associated with a poor prognosis for BC [4]. Hence, the identification of reliable molecular targets and targeted drugs for breast cancer patients to improve prognosis and treatment efficacy are urgently required.

The Hippo signaling pathway, which performs the main function of inhibiting cell proliferation and limiting organ overgrowth, is found in *Drosophila melanogaster* and is highly conserved in mammals [5]. YAP is the final effector of the Hippo pathway. When dephosphorylated, YAP is localized to the nucleus and binds to TEAD and acts as a transcriptional co-activator. YAP-TEAD mediates the expression of different oncogenes including those that regulate the tumor cell cycle, epithelial mesenchymal transition, migration, invasion, and chemoresistance [6]. Gene mutations and changes in the expressions of essential Hippo pathway components encourage the occurrence and development of breast cancer. Overexpression of YAP can significantly stimulate the proliferation of tumor cells [7]. Additionally, the expression of YAP has been found to be correlated with specific molecular subtypes of BC [8].

Clinically, Verteporfin (VP) has been utilized as a porphyrinic photosensitizer for the photodynamic treatment of neovascular macular degeneration for a long time [9]. Based on the previous research, non-photoactivated VP selectively binds to YAP and changes the YAP conformation process, thereby eliminating its interaction with TEAD [10]. In addition, VP blocks YAP function by increasing the level of 14–3-3σ protein in the cytoplasm [11]. Numerous studies have also shown that VP downregulated the transcription of YAP, which then inhibits cell proliferation-related genes, including cylinD1, cyclinE1, and TEAD in multiple tumor cells [12].

In this study, we assessed the influence and mechanism by which VP exerts its effects on the proliferation and apoptosis of Luminal A, Luminal B and Basal-like molecular subtypes of BC in relation to YAP. Our findings provide a novel theoretical basis for the use of VP in the treatment of BC.

## Methods

### Cell culture

Human Luminal A breast cancer cell line MCF-7, Luminal B breast cancer cell line BT-474 and Basal-like subtype cell line BT-549 [13] were bought from Zhongqiao Xinzhou Biology (Shanghai, China). MCF-7, BT-474 and BT-549 were maintained in RPMI-1640 (Hyclone, Logan, Utah, USA) supplemented with 10% FBS (Gibco, Carlsbad, CA, USA) and placed in a at 5% CO2 incubator at 37 °C. Verteporfin was bought from MCE (St Louis, USA).

### Cell viability assay

The MCF-7, BT-474 and BT-549 cells were added into 96-well plates at density of 4 × 104 cells per well. After incubation with VP, cell viability was assessed though CCK-8 assay (DOJINDO) at 24, 48, and 72 h. In brief, 10 μl of the CCK8 mixture was added into each well and the cells plates were placed in 37 °C incubator for 1.5 h. Absorbance was determined at a wavelength of 450 nm.

### Real-time PCR (RT-PCR)

Total RNA was extracted from VP-incubated cells utilizing the TRIzol regent (Invitrogen; Thermo Fisher Scientific, Inc.) using established protocol. cDNA was synthesized using the PrimeScript reverse transcription reagent kit (TIANGEN BIOTECH Co., Beijing, China) by following established guidelines. cDNA was assessed using qPCR on a SuperReal PreMix Plus (SYBR Green) system (TIANGEN BIOTECH Co.). The amplification conditions included: 95 °C for 15 min, 40 cycles of 95 °C for 20 s, 56 °C for 30 s and 68 °C for 30 s. The primer sequences used for qPCR were: YAP forward (F) 5′-TGACCCTCGTTTTGCCATGA-3′ and reverse (R), 5′-GTTGCTG CTGGTTGGAGTTG-3′; CTGF F, 5′-TGGAAGAGAACATTAAGAA GGGCA-3′ and R, 5′-TGCAGCCAGAAAGCTCAAAC-3′; AXL F, 5′-ACCCCAG AGGTGCTAATGGA-3′ and R, 5′-GTGGACTGGCTG TGCTTCC-3′; CYR61 F, 5′-GCAAGGAGCTGGGATTCGAT-3′ and R,5′-ATTCCAAAAACAGGGAGCCG-3′; GAPDH F, 5′-GCACCGTCAAGGCTGAGAAC-3′ and R, 5′-TGGTGAAGACGC CAGTGGA-3′. GAPDH functioned as the internal control. Gene expression was measured utilizing the 2-ΔΔCt method.

### Apoptosis assay

In order to determine the levels of cell apoptosis, the VP-incubated cells were washed and fixed at 4 °C with 4% paraformaldehyde for 30 min. After another wash, cells were incubated with 0.2% TritonX-100 for 15 min at room temperature, and cytometrically assessed using Principle In Situ Cell Death Detection Kit (Roche, Inc.), following established guidelines.

### Protein extraction and western blotting analysis

Total proteins were extracted utilizing RIPA lysis buffer that contained phosphatase and protease inhibitors. Protein concentration was evaluated utilizing a bicinchoniniacid assay (Solarbio, Beijing). The later steps of the western blotting analysis were conducted using standard protocol. Antibody directed towards GAPDH was acquired from Novus Biologicals, while antibodies directed towards YAP, TEAD, Bcl-2, BAX, p-YAP (Ser127), CYR61, CTGF, AXL, Caspase9, Cleaved Caspase9, PARP, and Cleaved PARP were bought through Cell Signaling Technology (Danvers, MA).

### Statistical analyses

Data were represented as mean ± standard deviation. Comparisons were conducted utilizing an unpaired 2-tailed Student t-test. The GraphPad Prism 5 software was utilized to assess statistical significance. *P* < 0.05 indicated statistical significance.

## Results

### YAP levels in different subtypes of BC cells

**Fig. 1:**
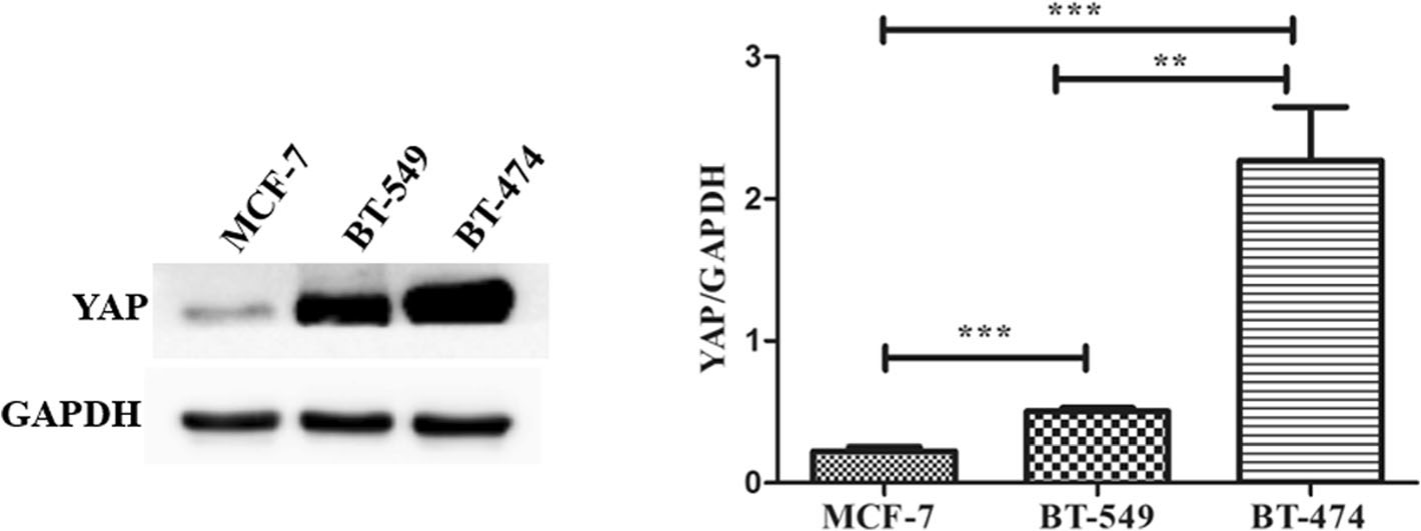
YAP expression in different subtypes of BC cells. Data is presented as mean ± SEM.*P < 0.05, **P < 0.01, ***P < 0.001

Western blotting analysis results showed that YAP was expressed in MCF-7, BT-474 and BT-549 cells. The expression of YAP in Luminal B BT-474 BC cells and triple negative breast cancer (TNBC) BT-549 cells were significantly higher than that in Luminal A MCF-7 cell. BT-474 cells showed the highest level of YAP expression among the three different subtypes of BC cell lines (*P* < 0.001) (Fig. 1).

### The effect of verteporfin on the proliferation of different molecular subtypes of BC cells

**Fig. 2:**
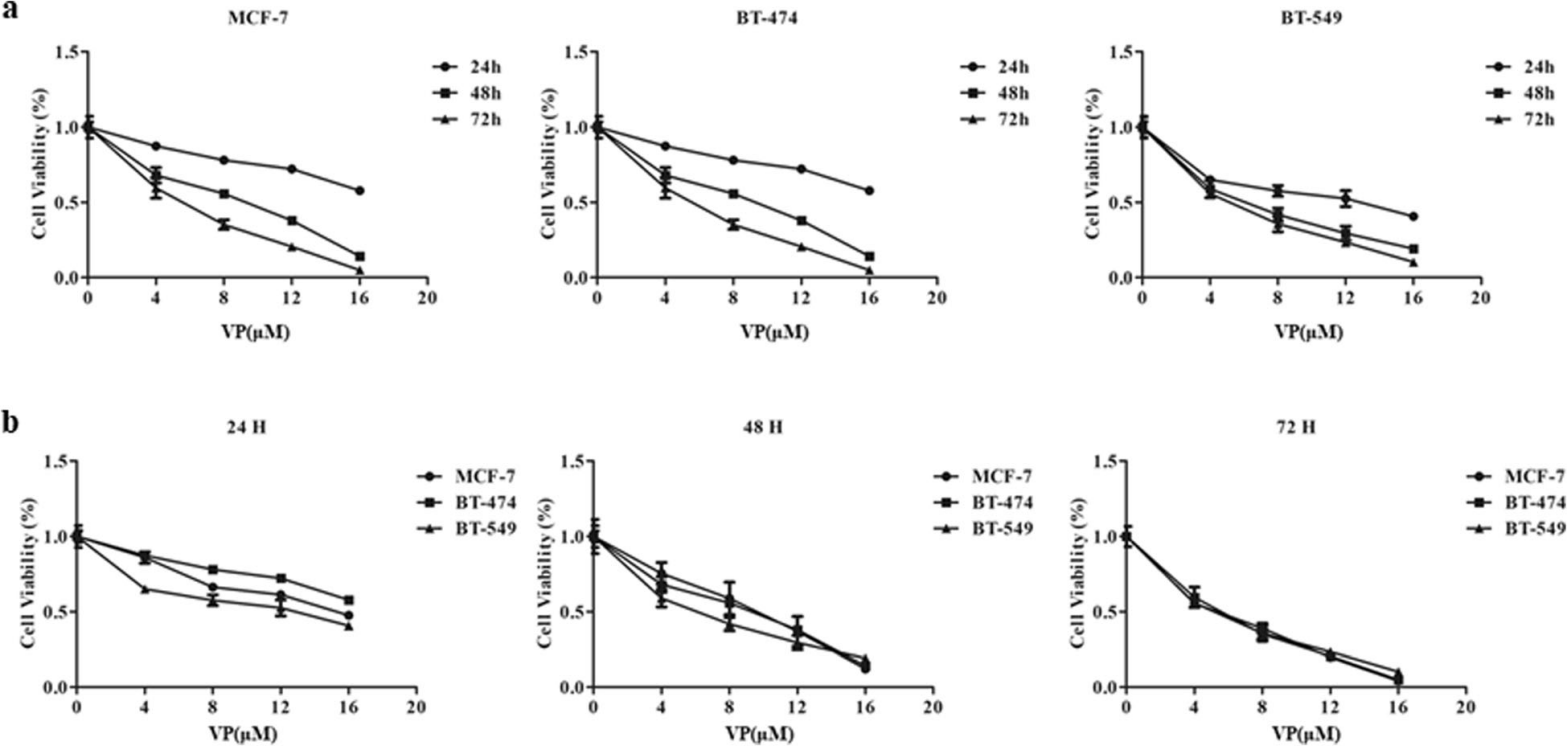
Verteporfin inhibited the proliferation activity of different subtypes of BC cell Lines. Different BC cells subtypes were treated using VP at different concentrations for 24, 48 and 72 h, at which time cell viability was determined (**a**, **b**)

Data from the CCK-8 test demonstrated that VP could suppress MCF-7, BT-474 and BT-549 cell growth in a dose-dependent manner, in comparison with the untreated MCF-7, BT-474 and BT-549 cells (Fig. 2a). Comparison between different molecular subtypes of BC cells under the same drug concentration found that after 24 h of VP treatment, the proliferation of BT-549 cells decreased most significantly. After treatment with 8 and 16 μm VP for 48 h, there was a statistically significant difference in the proliferation rate of different BC subtypes as compared with untreated cells (*P* < 0.05) (Fig. 2a). Altogether, VP decreased proliferation in all cell lines within 48–72 h compared with untreated cells.

### Verteporfin regulates the expression of YAP, TEAD and YAP-TEAD downstream targets

**Fig. 3:**
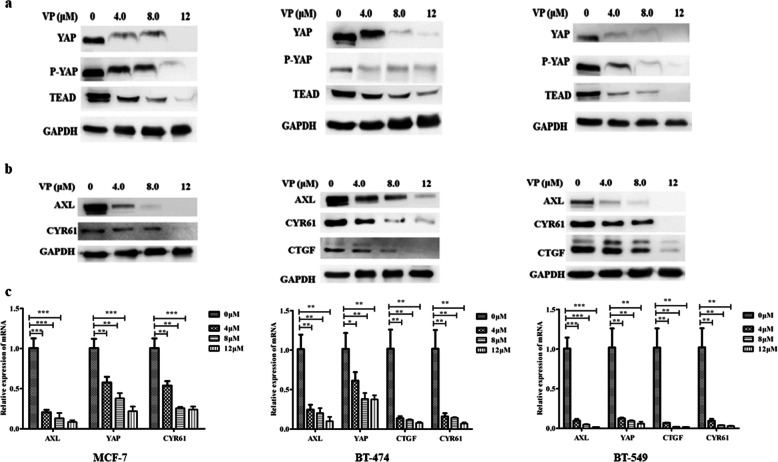
Verteporfin inhibits the expression of YAP, p-YAP and YAP downstream targets via disrupting YAP-TEAD interaction. Different subtypes of BC cells were treatment with different doses of VP for 72 h, and underwent western blotting to detect YAP, p-YAP and TEAD expressions (**a**), AXL, CYR61 and CTGF expressions (**b**), and Hippo pathway expressions (YAP, AXL, CYR61 and CTGF) (**c**)

As shown through the results of the Western blotting analysis, VP blocked YAP, p-YAP and TEAD protein expression in MCF-7, BT-474 and BT-549 cells (Fig. 3a). As the concentration of VP increased, the expressions of AXL and CYR61 proteins in different subtypes of breast cancer cells were downregulated. In addition, VP inhibited the expression of CTGF in BT-474 and BT-549 cells (Fig. 3b). VP also inhibited YAP-TEAD transcription in MCF-7, BT-474 and BT-549 cells in vitro. The RT-PCR results show that, in comparison with the controls, YAP, AXL and CYR61 mRNA expression levels in MCF-7, BT-474 and BT-549 cells decreased significantly after treatment with 4, 8 and 12 μM VP. Treatment with VP also downregulated the mRNA expression of CTGF in BT-474 and BT-549 cells (Fig. 3c). In brief, VP decreased the expression of YAP-TEAD downstream targets by inhibiting the expression of YAP and TEAD.

### Verteporfin induces cell apoptosis by disrupting YAP-TEAD interaction

**Fig. 4:**
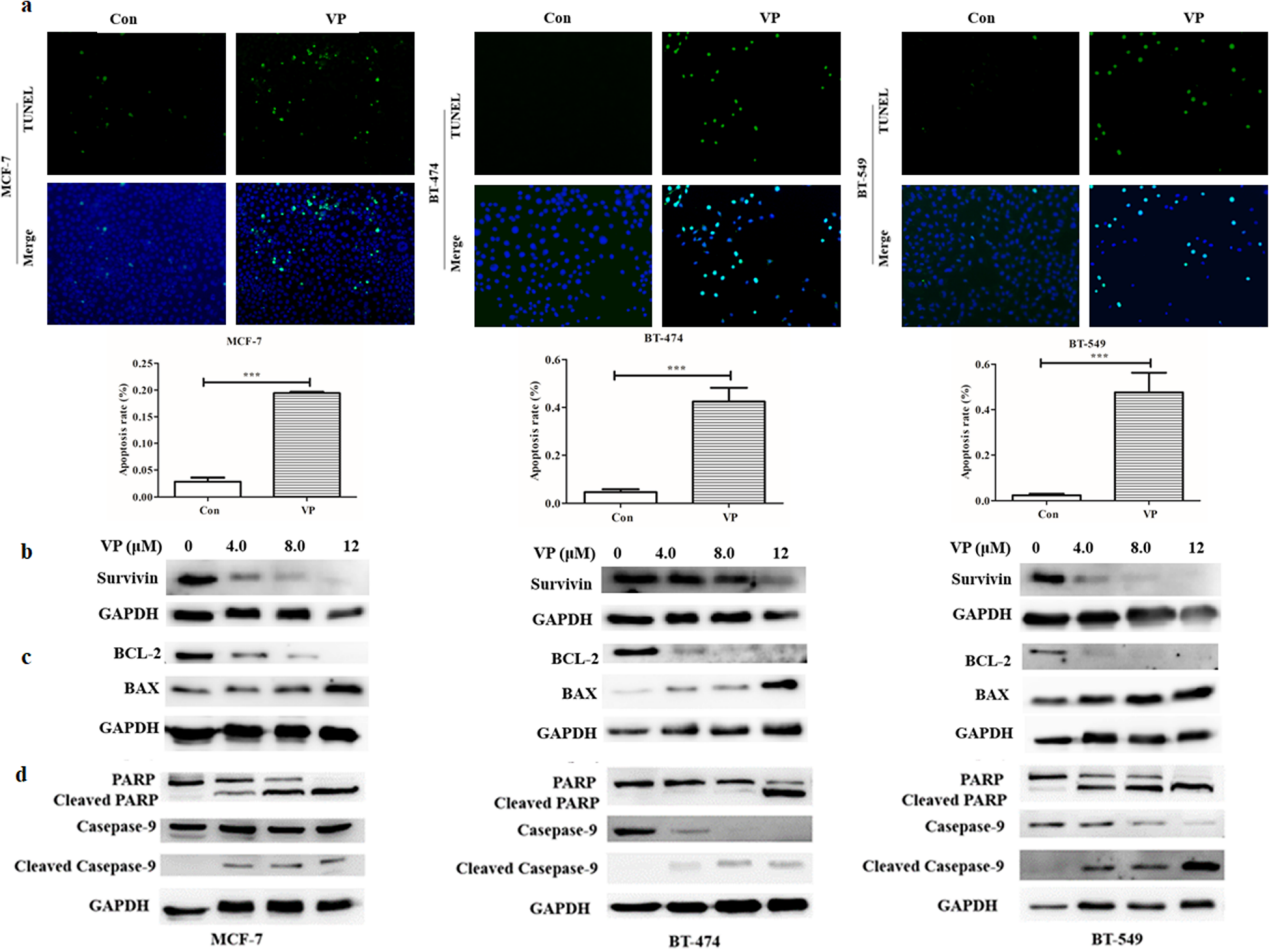
Verteporfin induces cell apoptosis in three different molecular subtypes of BC cells. BC cells were supplemented using the vehicle control or verteporfin and labelled with TUNEL. Data were presented as mean ± SD, *P < 0.05, **P < 0.01, ***P < 0.001 (**a**). BC cells were treated using different concentrations of verteporfin for 72 h, and subjected to western blotting analysis to determine the levels of the YAP target Survivin (**b**), BAX and Bcl-2 levels (**c**), and PARP, cleaved PARP, Caspase-9 and cleaved Caspase-9 expressions, full-length blots are presented in Supplementary Figure 4d (**d**)

TUNEL experiments showed that VP induced apoptosis in MCF-7, BT-474 and BT-549 cells (Fig. 4a). The western blot data indicated that treatment with VP led to the apoptosis of different subtypes of BC cells through decreased expression of the YAP downstream target gene Survivin (Fig. 4b), Bcl-2 and the ratio of Bcl-2/BAX, compared with the controls. Additionally, VP treatment led to an increase in levels of BAX (Fig. 4c), cleaved Caspase-9 and cleaved PARP proteins (Fig. 4d). These results demonstrated that VP, an inhibitor of the Hippo YAP signaling pathway, was highly conducive for inducing apoptosis among different molecular subtypes of BC cells by upregulating levels of cleaved Caspase-9, cleaved PARP, and downregulating the ratio of BAX/Bcl-2.

## Discussion

BC is the leading malignant type of tumor among females worldwide. In order to identify treatment targets for BC, it is important to analyze the genetic differences between normal and tumor cells. BC can be separated into five subclassifications: Luminal A, Luminal B, HER-2 overexpression, Basal like type and Normal breast-like subtype. The expression of PR and HER-2 can be used as the further indicators for the differentiation between Luminal A and Luminal B [14, 15]. Although the efficacy of clinical treatment methods for BC have progressed greatly, there are still only a limited number of treatment options for patients who experience chemotherapy resistance and metastasis [16]. Moreover, TNBC, which has been described using the deficiency of ER, PR, and HER-2, has been found to be associated with the worst prognosis due to increased rates of recurrence and a lack of effective targeted therapies among all major subtypes of BC [17].

Hippo is a complex tumor regulation pathway, and its deregulation has been found to be associated with changing normal state into a pathological cancer state. As the main effector of Hippo, YAP is phosphorylated by the Hippo Core complex and degraded in the cytoplasm [6]. When the Hippo upstream pathway is suppressed, hyperactivation of TEAD as a transcription factor requires the involvement of YAP as a coactivator to mediate downstream target gene transcriptional activity. Thus, YAP is translocated into the nucleus and promotes proliferation, metastatic development, and stem cell maintenance of cancer cells [18]. Knockdown of AXL, one of the YAP-TEAD target genes, decreases the growth and invasiveness of tumor xenographs [19]. Similarly, dysregulation of CTGF and CYR61 are strongly correlated with development of BC, prostate cancer and malignant melanoma [20–22]. Survivin plays a key role in controlling cell apoptosis, cell cycle and drug resistance [23, 24]. All of these functions depend on the interaction between YAP and TEAD transcription factors. Therefore, it can be observed that YAP exerts its oncogenic function by combining with the transcription factor TEAD to encourage the expression of genes that play a role in the progression and metastasis of cancer.

Several studies have demonstrated that VP decreased transcriptional activity of YAP by competitively binding to YAP and abrogating the interaction between YAP with TEAD, which blocks YAP-TEAD-stimulated tumorigenesis [12]. Mutations and the subsequent altered expression of YAP promoted the proliferation, invasion, and chemotherapy resistance of breast cancer cells. YAP plays a pivotal role in the proliferation, invasion, and chemotherapy resistance of breast cancer cells. YAP deficiency decreased lung metastasis ability in a breast cancer mouse model [25, 26]. Disruption of the interaction between YAP and TEAD not only weakened the expression of YAP downstream target genes, but also inhibited the proliferation, EMT and oncogenic transformation ability of many tumor cells. The YAP-TEAD complex is the last step in the transcriptional activity of YAP in the Hippo pathway, which caused the targeted blockade of YAP-TEAD by VP on the upstream proteins of the Hippo pathway and minimal side effects were expected. Our data demonstrated that VP inhibited the growth of the different subtypes of BC cells, and VP treatment efficiently prevented YAP-TEAD transcriptional activity. CCK8 analysis revealed that the TNBC BT-549 cell line was the most sensitive to the inhibitory effect caused by VP on cell proliferation. VP treatment led to YAP, AXL, CYR61 and/or CTGF downregulation in the BC cell lines of different subtypes, suggesting that these effectors may act as their own target genes as described in TNBC MDA-MB-231 cells, VP could inhibit the transcriptional activity of TEAD, reduce the protein expression levels of YAP target genes, AXL and CTGF, inhibit the migration, and induce the apoptosis of paclitaxel resistant MDA-MB-231 cells [27–29]. The results of western blotting analysis proved that the protein and mRNA expressions of YAP, as well as of YAP targeted genes decreased along with the increase in the concentration of VP. Compared with the untreated group, the expression levels of YAP, TEAD and Survivin in BT-474 cells decreased after 4 μM VP treatment (*P* < 0.05). However, when compared with the untreated group, the expression levels of YAP, TEAD and Survivin in MCF-7 and BT-549 cells at 4 μM VP treatment had decreased significantly reduced (*P* < 0.01). Similarly, at 4 μM VP treatment, the expression of YAP mRNA in BT-474 cells were downregulated, compared with that of the untreated group (*P* < 0.05), while the expression of YAP mRNA levels in MCF-7 and BT-549 cells showed a statistically significant difference, compared with the untreated group (*P* < 0.01).

Recently, studies have shown that non-photoactivated VP could induce the apoptosis of melanoma and pancreatic cancer cells through the activation of apoptosis-related proteins caused by preventing YAP-TEAD interaction [30–32]. Our results have demonstrated that VP induced the apoptosis of MCF-7, BT-474, and BT-549 cells by upregulating levels of BAX, cleaved Caspase-9, and cleaved PARP, and downregulating the level of Bcl-2. Furthermore, our western blotting analysis results indicated that the expression levels of cleaved PARP in MCF-7 and BT-549 cells were statistically different (*P* < 0.05) compared with the untreated group. Nevertheless, in BT-474 cells, no significant difference was found in cleaved PARP levels, compared with the untreated group (*P* > 0.05). Moreover, compared with the untreated group, there was a significant difference of in the expression of cleaved PARP in MCF-7 and BT-474 cells (*P* < 0.01) at 8 μM VP treatment, and a similar result was found in BT-474 cells (*P* < 0.05).

The studies of VP in breast cancer mainly focus on TNBC MDA-MB-231 cells, VP (1 μmol/L) inhibits the growth of the paclitaxel-resistant breast cancer cell line MDA-MB-231 [28], VP (10 μM, 3 h) reduced the expression of YAP in the ZNF367-overexpressing breast cancer MDA-MB-231 and 4 T1 cells, and significantly reduced lung metastases in mouse models [33], and sensitizes the HER-2 positive breast cancer cell line HCC1569 to lapatinib [34]. The purpose of this study was to explore the effect of VP on different subtypes of BC and its mechanism by targeting YAP gene under the condition of non-photoactivation. On the basis of existing data, we further supplemented the effect of VP on TNBC BT-549 cells, Luminal A MCF-7 cells and Luminal B (ER+, PR+, HER-2+) BT-474 cells in vitro. Meanwhile, we found that YAP was highly expressed in BT474 cells, which may increase the hope of targeted therapy for Luminal B and HER2+ patients besides endocrine and anti-HER2 therapy. These encouraging results suggested that the use of VP may be a promising strategy for the treatment of BC.

Experimental verification of various tumors indicated that targeting the Hippo pathway is an effective method of treating cancers [6]. Proteins of the CCN (CTGF/CYR61/NOV) family exhibit different levels of expression and transcription in different tumor tissues. Changes in the transcriptional activity of CCN have been found to be extremely important during tumorigenesis. CCN intervenes in embryonic development, angiogenesis, tumor heterogeneity and progression through various signaling pathways, including the Hippo pathway [35, 36]. Genetic studies have indicated that a stable level of CYR61 in MCF-7 cells could significantly counteract paclitaxel-induced apoptosis and increase chemotherapy resistance to doxorubicin [37]. In our study, we found that the treatment with VP downregulated the expression of CYR61 in Luminal A MCF-7 cells suppressing the proliferation of MCF-7 cells and providing a new treatment strategy for Luminal A endocrine patients. Our study found differential expression of CTGF in the three subtypes of BC. Interestingly, high level of expression of CTGF was observed in BT-549 cells, while CTGF expression was not detected in MCF-7 cells, which was concordant with the results of previous studies [38]. The activation of CTGF and CYR61 may exert divergent roles on the biological processes of different subtypes of BC.

Although based on theoretical concepts, there are still several problems with the use of VP as an inhibitor of YAP/TEAD interaction, VP is a photosensitizer drug that is already in clinical use. Cell exposure must be strictly controlled to avoid exposure to light. At present, no studies have declared the effects of YAP caused by photoactivated VP. Furthermore, the exact mechanism by which VP is able to affect the Hippo signaling pathway still needs to be evaluated in greater depth. More importantly, our study may pave the way for further drug development for BC.

## Conclusions

In conclusion, our data demonstrated that VP treatment inhibited the proliferation of MCF-7, BT-474 and BT-549 cells, with the main difference observed during short treatment periods (24 h) was that TNBC BT-549 cell lines responded faster than the others. VP also induced cell apoptosis by disrupting YAP-TEAD interaction, which led to a decrease in Survivin levels and the Bcl-2/BAX ratio, and cleavage of PARP and Caspase-9 in Luminal A MCF-7, Luminal B BT-474 and TNBC BT-549 cells in vitro. Repositioned VP targeting of YAP-TEAD activity may provide a theoretical basis for the development of VP as a novel targeted therapy, adjuvant therapy and precision treatment for breast cancer.

## Supplementary Information

**Additional file 1.**

**Additional file 2.**

**Additional file 3.**

## Data Availability

The datasets used and/or analysed during the current study are available from the. corresponding author on reasonable request.

## Abbreviations

ER: Estrogen receptor
PR: Progesterone receptor
HER-2: Human epidermal growth factor receptor-2
BC: Breast cancer
TNBC: Triple negative breast cancer
