# Supplementary figures and images for: Verteporfin inhibits cell proliferation and induces apoptosis in different subtypes of breast cancer cell lines without light activation
Source: BMC Cancer. 2020 Oct 29;20:1042. doi: 10.1186/s12885-020-07555-0 (PMC7599100; doi:10.1186/s12885-020-07555-0)

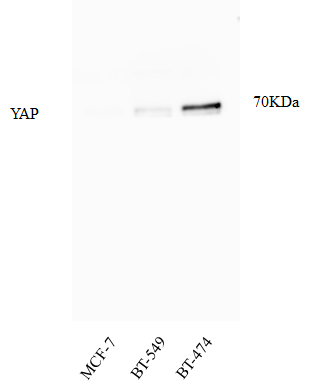

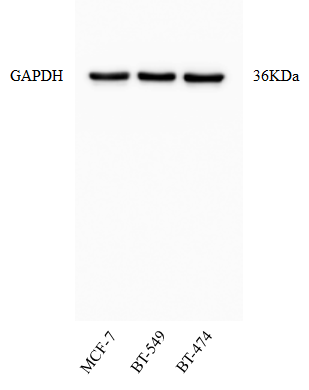

Supplement: Supplementary file 1 — Additional file 1. [file 12885_2020_7555_MOESM1_ESM.docx]

**MCF-7 3a**

**
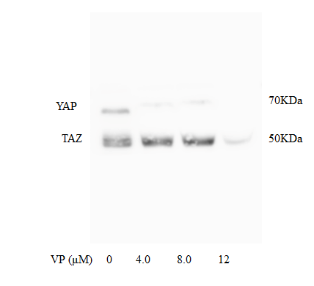

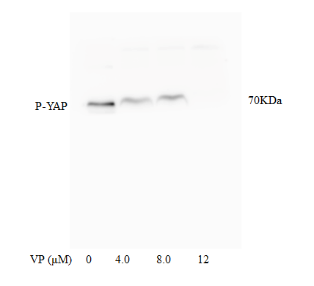

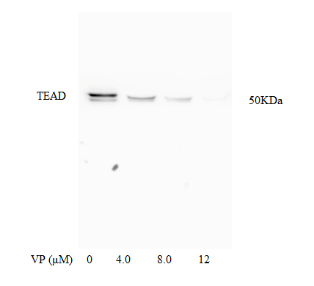

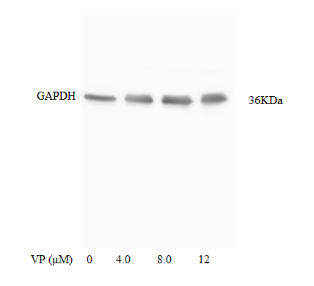
**

**BT-474 3a**

**
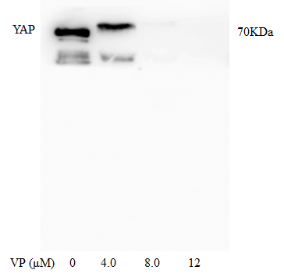

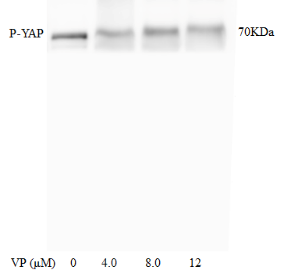

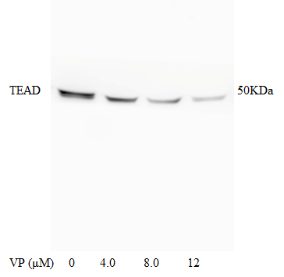

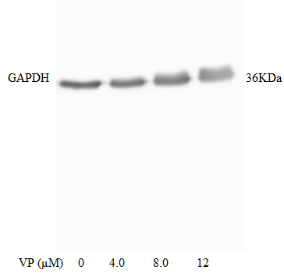
**

**BT-549 3a**

**
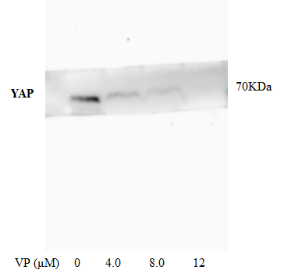

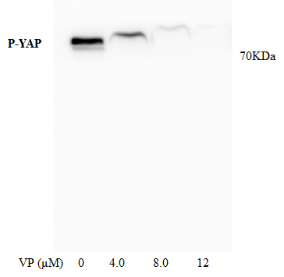

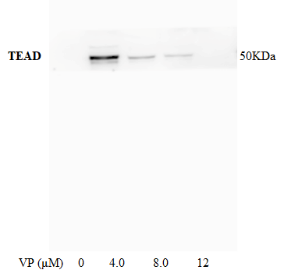

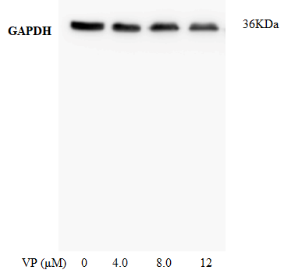
**

**MCF-7 3b**

**
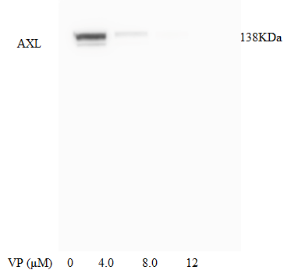

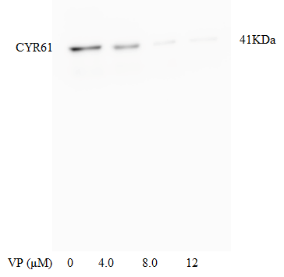

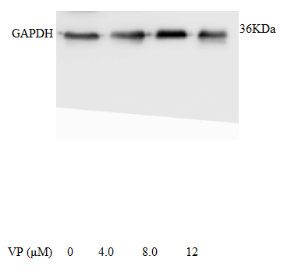
**

**BT-474 3b**

**
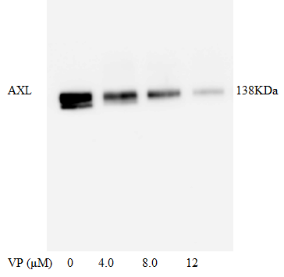

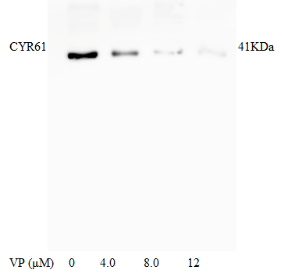

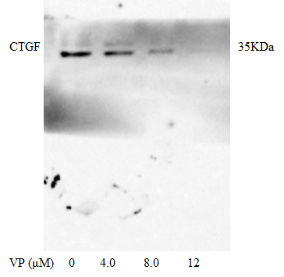

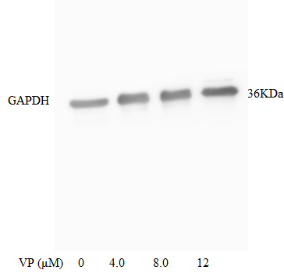
**

**BT-549 3b**

**
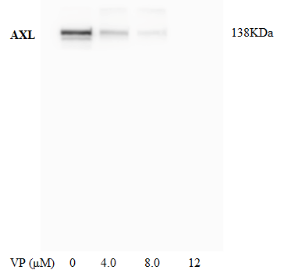

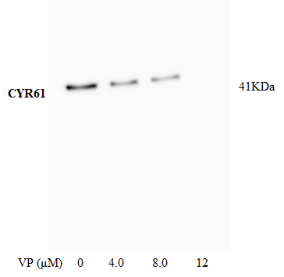

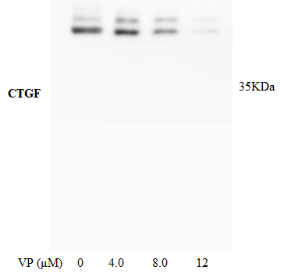

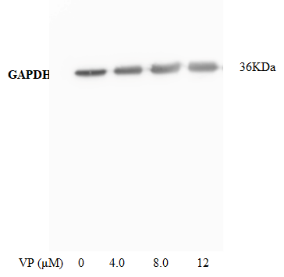
**

Supplement: Supplementary file 2 — Additional file 2. [file 12885_2020_7555_MOESM2_ESM.docx]

**MCF-7 4b**

**
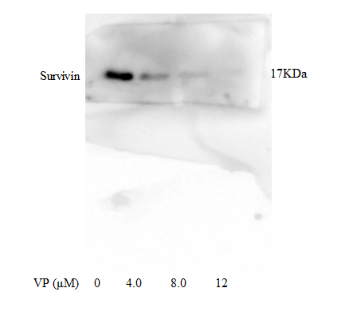

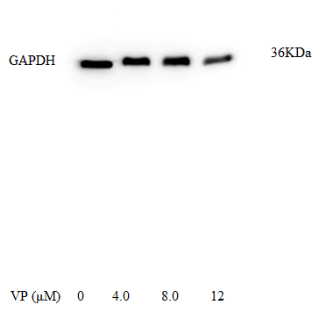
**

**BT-474 4b**

**
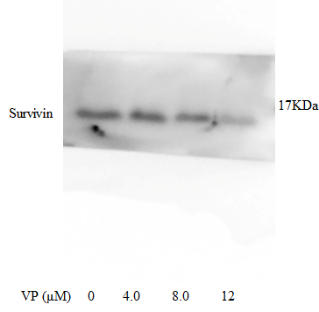

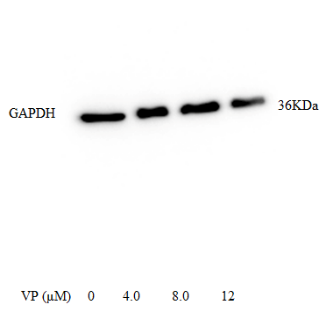
**

**BT-549 4b**

**
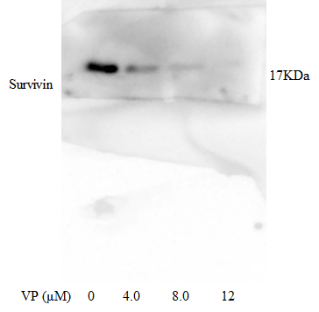

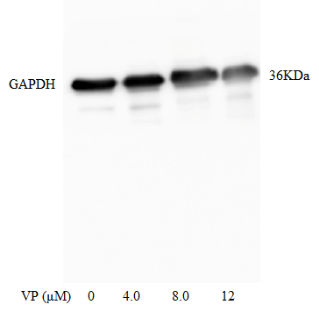
**

**MCF-7 4c**

**
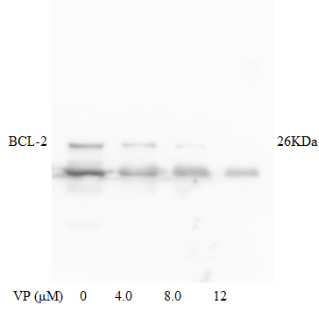

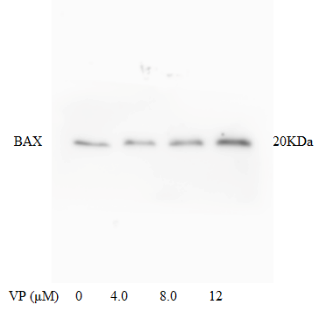

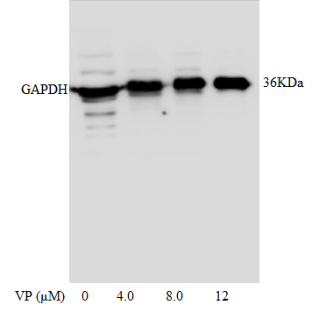
**

**BT-474 4c**

**
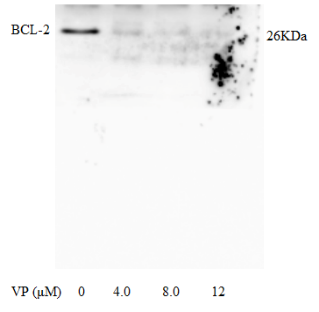

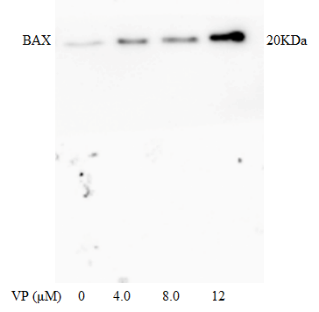

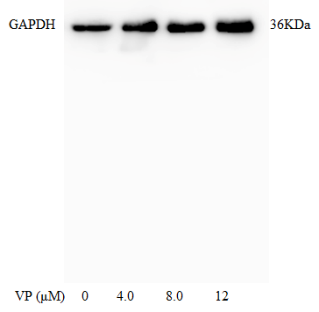
**

**BT-549 4c**

**
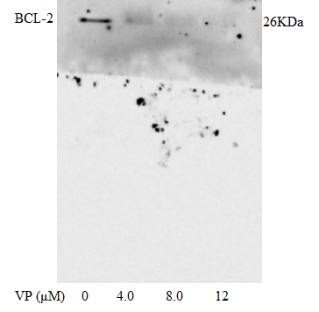

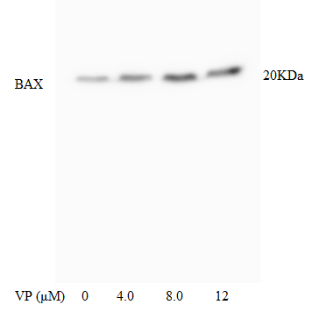

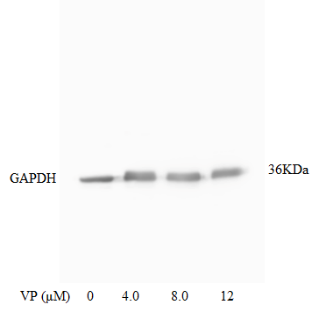
**

**MCF-7 4d**

**
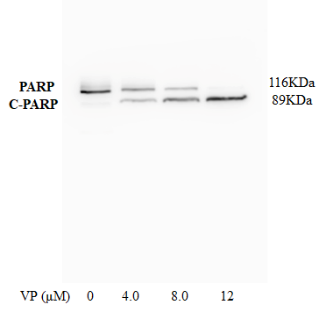

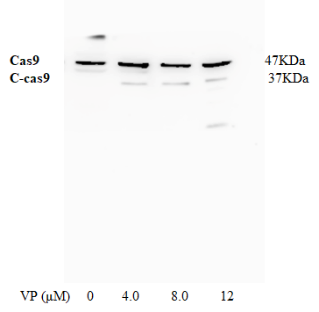

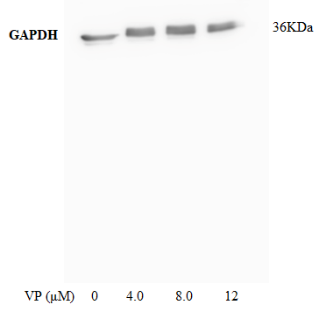
**

**BT-474 4d**

**
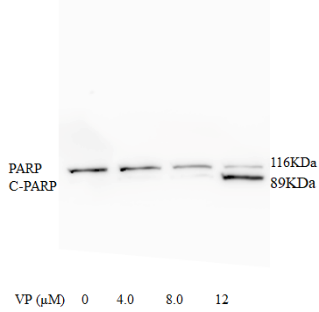

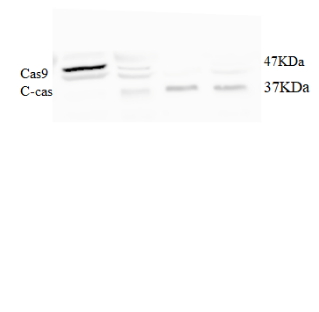

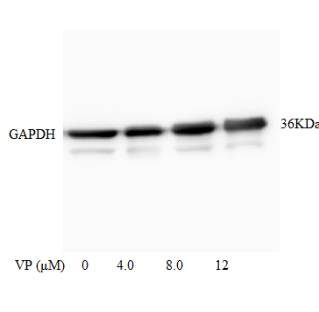
**

**BT-549 4d**

**
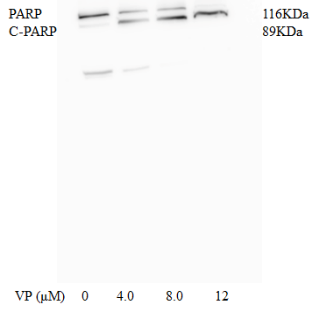

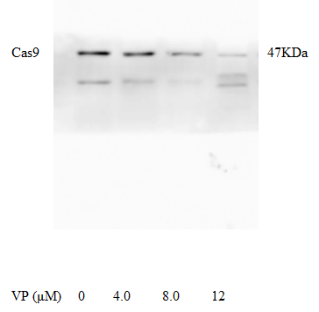

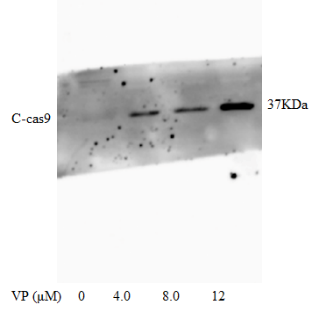

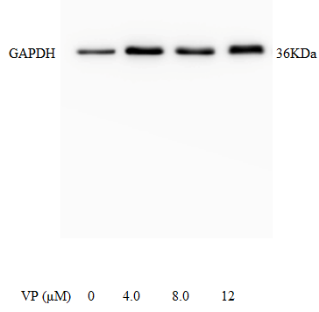
**

Supplement: Supplementary file 3 — Additional file 3. [file 12885_2020_7555_MOESM3_ESM.docx]
